# Biotechnological Applications of Transglutaminases

**DOI:** 10.3390/biom3040870

**Published:** 2013-10-22

**Authors:** Natalie M. Rachel, Joelle N. Pelletier

**Affiliations:** 1 Département de Chimie, Université de Montréal, 2900 Boulevard Edouard-Montpetit, Montréal, Québec, H3T 1J4, Canada; E-Mail: natalca.rachel@gmail.com; 2 CGCC, the Center in Green Chemistry and Catalysis, Montréal, H3A 0B8, Canada; 3 PROTEO, the Québec Network for Protein Function, Structure and Engineering, Québec, G1V 0A6, Canada; 4 Départment de Biochimie, Université de Montréal, 2900 Boulevard Edouard-Montpetit, Montréal, Québec, H3T 1J4, Canada

**Keywords:** biocatalysis, transglutaminase, protein modification, protein labeling

## Abstract

In nature, transglutaminases catalyze the formation of amide bonds between proteins to form insoluble protein aggregates. This specific function has long been exploited in the food and textile industries as a protein cross-linking agent to alter the texture of meat, wool, and leather. In recent years, biotechnological applications of transglutaminases have come to light in areas ranging from material sciences to medicine. There has also been a substantial effort to further investigate the fundamentals of transglutaminases, as many of their characteristics that remain poorly understood. Those studies also work towards the goal of developing transglutaminases as more efficient catalysts. Progress in this area includes structural information and novel chemical and biological assays. Here, we review recent achievements in this area in order to illustrate the versatility of transglutaminases.

## 1. Introduction

Harnessing the catalytic properties of enzymes is a field of research that continues to receive increasing attention. One of the most attractive characteristics of biocatalysts is that they are often highly chemo-, regio-, and stereo-selective. This provides potential for highly specific chemical transformations of complex, functionalized molecules. Additionally, biocatalysts are non-toxic, degradable, and functional in aqueous media at moderate temperatures and pressure, making them of high interest in the development of environmentally respectful synthetic methodologies. Due to these desirable properties, chemists are increasingly incorporating enzymes into their reaction schemes.

**Figure 1:**
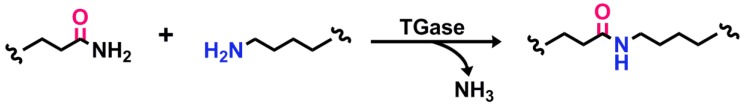
Amide bond formation catalyzed by TGase. Peptide- or protein-bound glutamines and lysines serve as substrates, releasing ammonia in the process.

The synthesis of amide bonds has the potential to benefit greatly from biocatalysis. The high stability of the amide functionality makes it one of the most favorable and commonly used in organic synthesis [1]. Some examples of compounds containing biocatalyzed amide bonds are found in the large-scale production of Atorvastatin (commercialized as Lipitor™), Nylon, penicillin, and aspartame. The high activation barrier to amide-bond formation is synthetically challenging; further development of biocatalysts for formation of a broad range of compounds remains of interest. Transglutaminases (TGases) are a family of enzymes (EC 2.3.2.13) that catalyze an acyl-transfer reaction between the γ-carboxamide group of a protein- or peptide-bound glutamine and the ε-amino group of a lysine residue, resulting in the formation of a relatively protease-resistant isopeptide bond [2] (Figure 1). TGases, having evolved to catalyze the formation of amide bonds with little competition from the reverse hydrolytic reaction, are a promising biocatalytic alternative to classical organic chemistry for amide bond synthesis.

TGases have been identified in many different of taxonomic groups, including microorganisms, plants, invertebrates, and mammals [3]. With respect to application, the vast majority of research has been done on two forms of the enzyme: the first is a calcium-dependant TGase found in tissues of animals and humans, referred to as transglutaminase 2 (TG2). TG2 is implicated in a number of physiological roles including endocytosis, cell-matrix assembly, apoptosis, and cellular adhesive processes [4,5,6]. There is much interest in studying TG2 from a medical standpoint to better understand its role in disease, including cataract formation [7], celiac sprue [8], and psoriasis [9]. The second enzyme is a calcium-independent, microbial transglutaminase (MTG), which was first isolated from *Streptomyces mobaraense* [10] and has since been isolated from other microbial strains, including, but not limited to, *S. griseocarneum*, *S. hygroscopicus*, and *B. subtilis* [11,12]. Both types of TGases have been studied extensively in academia and industry. Mechanisms for the reaction catalyzed by both TGase types have been proposed. The catalytic triad characteristic to cysteine proteases is present in the human factor XIII TGase (Cys314, His373, and Asp396) [13]. These residues correspond to Cys276, His334, and Asp358 in the highly conserved active site of guinea pig TG2 [14]. In the proposed mechanism, the cysteine and the histidine residues are principally involved in the acyl transfer reaction, where the aspartic acid residue hydrogen bonds with the histidine, maintaining a catalytically-competent orientation. The crystal structure of MTG revealed that this triad is not conserved; rather, it was proposed that MTG uses a cysteine protease-like mechanism in which Asp255 plays the role of the histidine residue in factor XIII-like TGases [15].

Of the two, MTG is more robust, and is commonly employed as a tool in the food industry to catalyze the cross-linking of meat, soy, and wheat proteins to improve and modify their texture and tensile properties [11,16]. Despite the medical importance of TG2 and widespread industrial use of MTG, many properties such as ligand binding, catalytic mechanism, and function in health and disease remain poorly understood, ultimately hindering further successful integration of these enzymes into novel applications and processes. Nonetheless, researchers are continually looking for ways to exploit the cross-linking activity of TGases for novel applications outside of the fields of human physiology and the food industry. Examples include tissue engineering [17], as well as textile and leather processing [18]. These applications generally utilize TGase to serve the same purpose it does in the food industry: non-specific protein cross-linking to provide improved physical and textural properties. A recent example involved increasing the mechanical strength of amniotic membrane, for applications in regenerative medicine [19]. The advances made in these fields have been covered in recent reviews [20,21], and will not be discussed in detail here. This review focuses on recent advances made in studying TGases in the scope of biotechnology and characterization, including advances in assay development, site-specific modification of biomacromolecules, and protein labeling.

## 2. Production and Engineering of TGases

### 2.1. Transglutaminase Expression and Purification

Both TG2 and MTG are readily recombinantly expressed and purified in bacterial hosts [22,23]. Using these methods, the production of TG2 in a hexa-histidine labeled form has become routine [22,24,25], although other forms of TG2 can remain a challenge to obtain in good yield. A complementary technique for the purification of hTG2 was recently reported, in which hTG2 was expressed as a fusion with glutathione S-transferase (GST) and followed by a one-step affinity chromatography purification [26]. Unlike TG2, the purification of the most widely used MTG (from *S. mobaraensis* and homologs) is complicated by the fact that the native enzyme is expressed as a zymogen (pro-MTG); a 46-residue *N*-terminal pro-sequence must be proteolytically cleaved in order for MTG to be rendered functional. There are reports of other MTGs that can be directly expressed as recombinant, active enzymes [27,28], however these are not as well characterized. Three solutions to this problem have been reported: (1) expression of pro-MTG followed by *in vitro* activation using a protease [29,30]; (2) direct expression of insoluble MTG lacking its *N*-terminal pro-sequence (mature MTG) followed by refolding [23], or (3) co-expression of pro-MTG with the activating protease in *Streptomyces* [31] or *E. coli* [32]. Each of these strategies has limitations: the first strategy can achieve high yields and activity, but involves lengthy activation methodologies (N.M. Rachel and J.N. Pelletier, unpublished observations). The second often leads to a low expression or insoluble protein, while the third strategy can result in protein degradation, affecting the yield [33]. 

**Figure 2:**
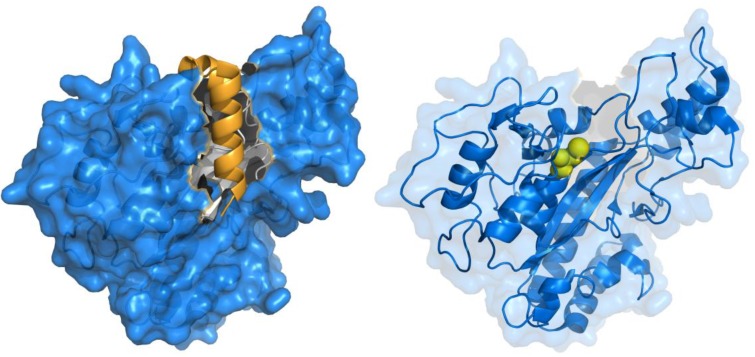
Crystal structure of MTG (PDB ID: 3IU0). The active site of the zymogen is covered (left) by an α-helix (gold), which is cleaved upon activation, exposing the active site cysteine residue (right, yellow spheres) that is critical for activity.

Recently, MTG from *S. hygroscopicus* was successfully produced in its active form in *E. coli* by simultaneously expressing the pro-sequence and mature MTG as separate polypeptides under the control of a single T7 promoter [34]. Expression of the pro-sequence prior to the mature MTG polypeptide was found to be essential for activity, as well as an *N*-terminal pelB sequence for periplasmic localization. This supports the hypothesis that the pro-sequence is required for proper folding and soluble expression of MTG. Improved efficiency of MTG maturation in *Streptomyces* was also recently reported, by engineering more protease-labile linkers into the pro-propeptide [35]. The structural basis for this requirement can be understood upon observing the crystal structure of pro-MTG, which was determined at 1.9-Å resolution [36] (Figure 2). The pro-sequence folds into an α-helix, covering the putative active site cleft by adopting an L-shaped conformation. The active site cleft is predominantly composed of two flexible loop regions, explaining how the presence of this ordered helix stimulates proper folding, in a fashion similar to that of the pro-sequences for subtilisin BPN’ and other proteases [37].

Two biophysical studies focusing on the detailed mechanism of unfolding and refolding of MTG were reported by Suzuki and colleagues [38,39]. In the first, a two-step refolding process of acid-denatured MTG was proposed after probing the effect of pH and salt concentration. The authors then applied this protocol to pro-MTG in the second report, such that by partially unfolding the enzyme, the internal residues would be exposed when in the presence of a deuterated solvent. This solvent exposure is often necessary so that hydrogen back-exchange occurs for all residues in the protein, allowing for accurate measurements using nuclear magnetic resonance (NMR) spectroscopy to be taken. Complete back-exchanges for internal residues of pro-MTG were observed by NMR spectroscopy, and the authors were able to recover the properly folded form of both pro-MTG and mature MTG, reporting refolding yields of 84% and 40%, respectively.

### 2.2. Engineering TGases for Altered Function and Properties

The design of enzymes with improved or non-native properties has become a common approach [40,41,42]. Engineering TGases may provide solutions to increase their applicability in biocatalytic contexts. TG2 has been engineered towards catalyzing amide bond formation between various synthetic substrates, by altering its substrate specificity [43]. A model peptide substrate, benzyloxycarbonyl-L-glutaminylglycine (Z-QG), was modified to yield a fluorescent umbelliferyl ester derivative (Z-GU) in order to screen for variants of TG2 with altered transpeptidase activity. Two separate point mutations were identified, which broaden the substrate scope of TG2, resulting in variants that can accept threonine methyl ester. To the best of our knowledge, this remains the only study focused on evolving TG2, and so the efforts in this field remain largely conservative. 

With respect to MTG, logistical complications of expressing the mature enzyme and the lack of a simple, high-throughput screening assay remain major challenges for engineering. Nonetheless, enhancing the activity and thermostability of MTG has been probed by two different studies. Pietzsch and colleagues [44] performed random mutagenesis using a microtiter plate-based screening method adapted to the standard hydroxamate assay [45] to measure activity. A library of 5500 clones generated randomly by error-prone PCR was initially screened, 70 of which showed higher activity following incubation at 60 °C. Following another round of mutagenesis, the nine clones with the highest residual activity were further characterized. The single-residue variant Ser2Pro was found to have an optimal functioning temperature of 55 °C, an improvement of 5 °C compared to the native enzyme. More recent efforts using saturation mutagenesis and DNA-shuffling by the same group yielded a triply substituted variant of MTG exhibiting a 12-fold and 10-fold higher half-life at 60 °C and 50 °C, respectively [46], although the Ser2Pro variant remained the most active at 55 °C. Chen and colleagues also evolved thermostable variants of MTG by combining saturation mutagenesis and the deletion of various *N*-terminal residues [47]. The variant Del 1-4E5D, which lacks the first four *N*-terminal residues and substitutes the fifth residue, exhibits a modest 1.85-fold higher specific activity and a 2.7-fold higher half-life at 50 °C compared to the wild-type enzyme.

**Figure 3:**
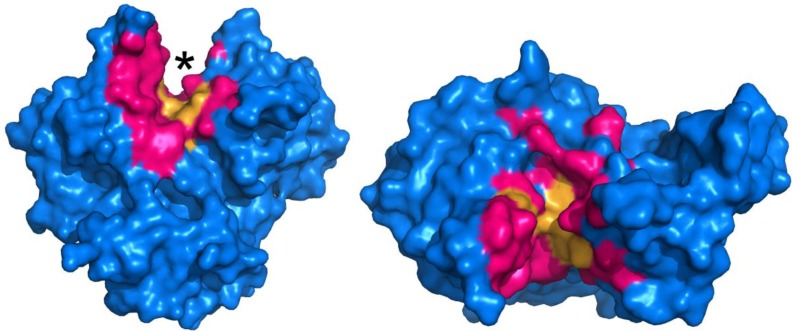
Surface representation of MTG (PDB ID: 1UI4), illustrating active site residues investigated by mutagenesis (pink and orange regions) [48]. The active site cleft is indicated by an asterisk. Residues in orange, upon substitution to alanine, resulted in activity of 5% or less than the wild type, revealing their importance.

Determining what residues to be the focus of mutagenesis is key to the success of any protein engineering initiative. In order to probe which residues may be necessary for MTG activity, an alanine screen of 29 residues that are either located in proximity to, or constitute the putative active site, was performed [48]. Docking and molecular dynamics simulations were also performed in order to propose the manner in which the model peptide substrate Z-QG binds to the enzyme, and the mutagenesis results were interpreted in the context of the docking results. The results suggest that an extended surface along the active site cleft is involved in binding of a protein substrate. Furthermore, it appears that a number of hydrophobic and aromatic residues are important for interacting with Z-QG, which is summarized in Figure 3. Despite this data, further evolution of TGases has yet to be reported.

## 3. Substrate Specificity

While the acyl-transfer reaction catalyzed by TGase between the peptide- or protein-bound glutamine and lysine substrates is well characterized, the preference the enzymes display towards a specific peptide sequence is not obvious. Most glutamine and lysine residues will serve as a substrate, with varying degrees of reactivity, as long as they are accessible to TGase [49]. This limits the application scope of TGases where reactivity towards a specific substrate is required, such as protein labeling. Ten years ago, highly-reactive glutamine-containing substrates for TG2 were reported, which in some cases are related to physiologically-relevant targets [25], and in other cases were empirically designed and contain more than one glutamine for increased reactivity [50]. The secondary structure surrounding the glutamine appears to be important in defining reactivity [25]. With respect to MTG, the native substrates and physiological function of the enzyme are not known. This has led researchers to approach the question of TGase’s poorly understood substrate preferences from two different perspectives. The first is to probe the specificity of the enzyme towards specific peptide or protein substrates of interest by analyzing which glutamine or lysine residues are reactive and to what degree. The second is to screen libraries of peptide sequences or other compounds with the goal of either identifying a preferred sequence pattern, or to identify highly reactive substrates. Recent advances with both of these approaches TGase substrate specificity offer further insight into the utility as well as the remaining limitations of these enzymes toward their biotechnological application.

The reactivity of MTG towards glutamine residues on several different proteins has been recently investigated. Using the sensitivity of mass spectrometry (MS), the identification of the glutamine residues most reactive towards MTG-catalyzed PEGylation was described [51]. In that study, a monodisperse Boc-PEG-NH_2_ was used as the amine substrate on three model proteins: granulocyte colony stimulating factor (GCSF), human growth hormone (hGH), and apomyoglobin (apoMb). The former two proteins were selected for their importance as therapeutic proteins, and apoMb for being a model protein regarding the investigation of protein structure, folding, and stability. Despite the fact that GCSF, hGH, and apoMb have 17, 13, and 6 glutamine residues, respectively, only one or two per protein were modified by MTG. All effectively PEGylated glutamines were within disordered regions, suggesting that a flexible polypeptide substrate facilitates binding of MTG to target glutamines. A similar study used type I collagen as a protein substrate [52]. The resulting intermolecular collagen cross-links were quantified by digesting the collagen sample and separating of the fragments by HPLC. No more than five cross-links were formed out of a maximum of 27 possible. At least half of the cross-links were located within the triple helical region of the collagen molecule; however, the specific residues that were modified by MTG were not identified. Importantly, the cross-links were introduced by MTG only after the collagen had been at least partially heat-denatured, supporting the correlation between structural disorder of the target and recognition by MTG. To further investigate the importance of secondary structure and MTG’s apparent preference for flexible polypeptide regions, the reactivity of MTG towards apoMb, α-lactalbumin (α-LA) and fragment 205-316 of thermolysin was analyzed [53]. These extensively studied proteins are models of α-helices, β-sheets and unstructured regions, respectively. Once more, despite many glutamine residues being present, few were substrates, with flexible or unstructured regions experiencing the highest reactivity. MTG discriminated notably less against protein-bound lysine as substrates, although those located in disordered regions were indeed more reactive. While this is by no means an exhaustive study of MTG’s substrate reactivity with respect to secondary structure, MTG’s reactivity towards flexible or unfolded regions for both glutamine and lysine protein substrates is further enforced.

Notwithstanding those advances, searching for superior glutamine recognition sequences that can be grafted onto a desired labeling target (often referred to as a “Q-tag”) requires a high-throughput methodology in order to screen varying glutamine-containing sequences in an efficient manner. This had been previously done by phage display [54,55], in which phage-displayed dodecapeptide libraries on the order of 10^11^ members were screened for reactivity toward TG2 and MTG. Regarding MTG, a preference for an aromatic amino acid *N*-terminal to the glutamine was observed, as well as for an arginine and a hydrophobic amino acid at the +1 or +2 positions. However, no clear preferred amino acid pattern was obvious among the results. Building on this data, sequences determined to be the most reactive were synthesized and tested as penta- and heptapeptide substrates [56]. The pentapeptides’ affinity for MTG were as low as Z-QG (in the range of 50 mM); however two heptapeptides, 7M42 (Ac-YELQRPY-NH_2_) and 7M48 (Ac-WALQRPH-NH_2_), were found to have a 4.5 and 19-fold decrease in K_m_, indicating that the identity of surrounding amino acids affect K_m_. Using a complimentary approach, the search for a Q-tag was expanded by recently employing mRNA display as a high-throughput screen [57]. Peptides that served as substrates became covalently bound via MTG reaction with hexa-lysine conjugated beads. Two pentapeptide sequences in particular were reported to have considerably higher reactivity and affinity for MTG (RLQQP and RTQPA), which vary considerably from the results obtained *via* phage display. In light of these results, valuable insight into the sequence and structural preferences for efficient TGase recognition of glutamine has been obtained. However, they do not yet converge onto a single, high-affinity Q-tag. The identification of a peptide sequence that is highly specific for MTG has also yet to be demonstrated, and so the precise requirements for selective glutamine binding to TGases remain under investigation.

The structural requirement of MTG’s amine (lysine) substrate has previously been suggested to be considerably less strict than that of its amide (glutamine) substrate [58,59,60]. Along the same line of thought, as with the glutamine substrate, a recent study used an *in vivo* Förster resonance energy transfer (FRET) quenching assay in order to screen for highly reactive lysine recognition sequences (“K-tag”) in *E. coli* [61]. The sequences screened were limited to pentapeptides with a lysine fixed at the center position. Although there was no repeated or consensus sequence determined by the screen, the pentapeptide KTKTN was found to be of reactivity comparable to a hexa-lysine tag. Synthetic amide and amine substrates were also previously tested for activity in order to determine if MTG could utilize non-natural substrates [62]. This was investigated in greater detail recently by screening amine compounds with increased diversity of chemical substituents and functional groups [63]. Overall, MTG was found to be highly promiscuous for its primary amine substrate, and amines attached to a less hindered carbon as well as amines with a longer hydrocarbon linker exhibited increased reactivity. Aromatic and small, polar amine-bearing compounds were observed to be excellent substrates as well. These studies help broaden the scope for modification of glutamine-containing peptides and proteins by TGases.

## 4. Assays

**Figure 4:**
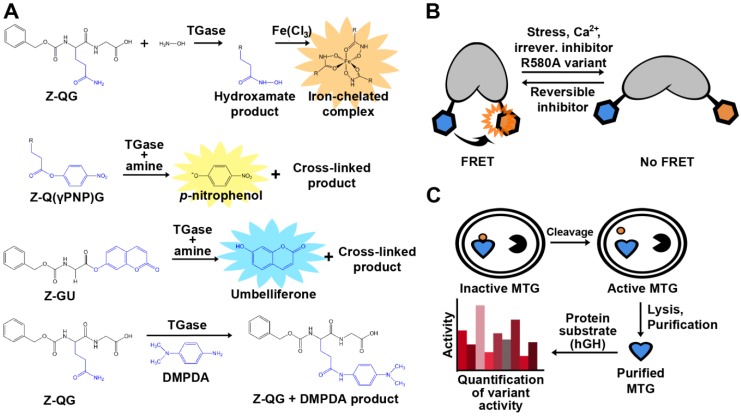
Examples of assays used for detection of TGase activity. (**A**) Colorimetric and fluorescent product release activity assays. The hydroxamate assay (top) remains the standard method to determine and compare TGase activity. TG2 activity can also be quantified by the release of *p*-nitrophenol (PNP; λ_max_ = 405 nm), umbelliferone (λ_em_ = 465 nm), or by the formation of an anilide product (λ_max_ = 278 nm) following conjugation with *N,N-*dimethyl-1,4-phenylenediamine (DMPDA). (**B**) Cartoon representation of the TG2 conformational FRET sensor. (**C**) *In vivo* activation of MTG allowing for in-cell assaying.

Assay development is key to the advancement of medicine, cell biology, and biotechnology. With respect to TGase, some goals for novel or improved assays include: the identification of highly specific substrates or inhibitors, higher sensitivity, cellular visualization in order to better understand the role of TGase in disease, and facilitation of TGase engineering by high-throughput screens. The detection of TGase activity is not immediately obvious due to the fact that none of its reactants or products absorb strongly at a distinctive wavelength, nor are they fluorescent. A standard end-point, colorimetric assay was developed early on (Figure 4A). The assay uses Z-QG as a model glutamine substrate and hydroxylamine as the amine substrate. The addition of TGase catalyzes the formation of an isopeptide bond and a hydroxamate group, and upon the addition of a concentrated ferric chloride solution, results in the development of a yellow color [45]. The hydroxamate assay remains in use to this day in order to determine kinetic constants, but its discontinuous nature and low molar absorptivity limit its applicability. As a result, a number of novel TGase assays have since been developed for use not only *in vitro*, but *in vivo* as well. Some colorimetric and fluorometric examples include sensitive assays involving the enzymatic release of *p*-nitrophenol, 7-hydroxycoumarin, and the production of chromophoric anilide [64,65,66].

An alternative approach has been to label a protein substrate of interest in a reaction mediated by TG2 with a biotinylated fluorophore and subsequently isolate the newly biotinylated protein with streptavidin beads, allowing for immobilization and separation of the product [67]. The sensitivity of this assay allows for detection of 0.6 mU purified TG2, and can also be applied to crude lysates, making it possible to screen for low transpeptidase activities. However, the sensitivity is less than that of assays using dansylcadaverine to detect product formation, which have been reported to detect as little as 60 μU [68] and 10.8 μU [69] of TG2. This fluorescent alkylamine is commonly used as a substrate for TGases to fluorescently label proteins, and removal of unreacted dansyl cadaverine may reduce background. To address this issue, magnetic dextran coated charcoal has been used to capture and magnetically sediment unreacted dansyl cadaverine, in a method readily adapted to 96-well plate format [69]. The first assay monitoring the change in fluorescence anisotropy has been recently described [70]. A fluorescein-labeled substrate peptide is monitored for an increase in fluorescence anisotropy as it is cross-linked to a significantly larger substrate, bovine serum albumin (BSA). The assay allows for detection of TG2 as low as 300 pM. The assay also detects product formation; however, a large difference in mass between substrates and product is required in order for detection to occur. Crystal structures of TG2 reveal that the enzyme undergoes a sizeable conformational change upon substrate binding [71]. In the presence of GDP/GTP, TG2 adopts a “closed” conformation that is inactive [72]. When bound to a substrate-mimicking inhibitor, TG2 was found to be in an “open” conformation, suggesting that the open conformation is the catalytically active form of the enzyme [72]. These conformational changes were recently used as a basis for novel activity assays of TG2. In the first assay, TG2 is used as a biosensor that allows for quantitative assessment in live cells using FRET, as measured by fluorescence lifetime imaging microscopy (FLIM) [73] (Figure 4B). This concept was further developed to monitor the real-time, ligand-induced conformational changes of TG2 using kinetic capillary electrophoresis, making this a rapid detection method [74]. As mentioned above, Kim and coworkers recently reported a FRET quenching assay to screen MTG activity in *E. coli* [61]. Each of the two peptide substrates is genetically fused to a fluorescent protein; if the peptide substrates are cross-linked upon exposure to TGase, a FRET quenching results. This approach is highly flexible in that it will allow library screening for either peptide substrate.

Previously, interest has been expressed to engineer TGases towards novel applications [43,44]. With regard to MTG, its requirement for activation complicates the development of a high-throughput screening assay. In effort to circumvent this obstacle, Zhao and co-workers demonstrated an *in vivo* selection assay for MTG [32] (Figure 4C). MTG was co-expressed with the 3C protease in order to activate the enzyme. The authors performed site-saturation mutagenesis on two different residues, Y62 and Y75, and used the assay to identify a variant that favors the conjugation of PEG to a specific glutamine (Q141) of human growth hormone. Two variants were found to be exclusively specific for Q141, even after 30 hours of reaction time. In order to determine activity, a previously established scintillation proximity assay was used [75], complexifying the methodology. A simple, continuous, colorimetric TGase assay was recently adapted in order to easily determine kinetic parameters of MTG with different substrates. Glutamate dehydrogenase activity was coupled to ammonia release upon deamination of the glutamine substrate for MTG, resulting in a decrease in NADH readily observed at λ_max_ = 340 nm [56]. 

## 5. TGases as Biocatalysts for the Production of Novel Biomaterials

The earliest biocatalytic use of TGases was in the food industry [11,16], which continues on a large scale to this day. Novel biotechnological applications have since been fostered to expand the biocatalytic utility of TGases outside of the food industry. Progress in this field has hastened in conjunction with recognition of their flexibility with respect to the primary amine substrate. This has helped open the door of possibilities with regard to covalently modifying protein- or peptide-bound glutamines with a wide array of compounds. The increasing diversity is welcomed: as previously discussed, a number of polymer-protein conjugates have been prepared with TGase using PEG to tailor the properties of the substrate protein to towards a more favorable therapeutic profile, such as enhanced stability and decreased toxicity. Recently, the polymer repertoire was expanded by synthesizing conjugates using hydroxyethyl starch [76]. It is a biodegradable alternative to PEG for commercial use as a blood plasma volume expander, potentially making it a more suitable polymer for protein conjugation. Taking this concept a step further, protein lipidation was demonstrated using MTG, with the goal of altering the behavior of the conjugated protein by controlling its localization via increased amphiphilicity [77]. Proteins can be regarded as biopolymers themselves, and can thus be assembled into larger biomolecular complexes in order to achieve altered functionality and properties. However, such a complex is only of use if its assembly can be controlled. A supramolecular protein complex, composed of *E. coli* alkaline phosphatase (AP) and streptavidin, was constructed with the aid of MTG [78]. The strong avidin-biotin interaction was exploited to direct the assembly of these two protein building blocks into a larger complex, by having AP site-specifically conjugated with biotin using MTG. The location of biotin conjugation on AP was crucial to create large structures and retain AP activity. Finally, MTG has also been found to be effective at modifying the structure of peptides containing a glutamine and lysine residue by cyclization [79]. 

Proteins and peptides are not the only biological molecules that have been modified using TGases; MTG has been recently used to site-specifically attach diverse compounds, at multiple positions, onto antibodies [80,81]. Glycosylation normally prevents TGase from effectively modifying antibodies, but the glycosylation pattern was modified such that MTG was able to react at specific locations. The resulting antibody-drug conjugates (ADCs) are of interest as potential therapeutic solutions, and tweaking their pharmokinetic properties by conjugation with different compounds may yield new therapeutic avenues that were previously unfeasible.

## 6. Protein Labeling

**Figure 5:**
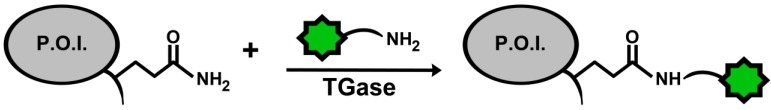
General scheme for protein labeling using TGase. The protein of interest (P.O.I.) carries an accessible glutamine residue, for TGase-catalysed reaction with an amine-substituted fluorophore; alternatively, the P.O.I. carries a reactive lysine residue for reaction with a glutamine-modified fluorophore.

A specific application of TGases that is gaining importance is their use as a tool to site-specifically label proteins with the goal of visualization within complex biological systems, such as in living cells. The typical strategy is to introduce an amide- or amine-containing fluorophore substrate into the system, along with TGase, to form an isopeptide bond with a specific lysine or glutamine, respectively, on the target protein (Figure 5).

**Figure 6:**
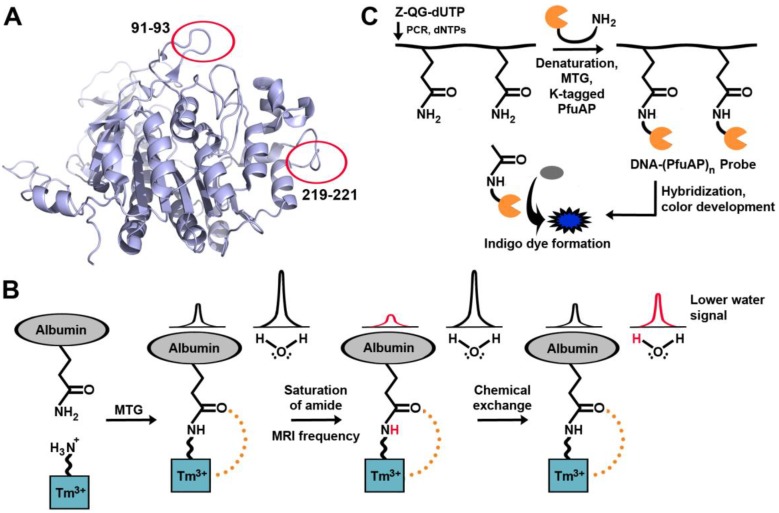
Examples of TGases applied for visualization of biomacromolecules. (**A**) Locations of independently encoded 13-mer peptidyl loop K-tags on bacterial alkaline phosphatase. (**B**) MTG-aided enzymatic detection of nucleic acids. (**C**) The paramagnetic agent is cross-linked to a glutamine, generating the CEST effect. Magnetic resonance saturation is transferred to water following saturation of the amide proton.

A fluorescent analog of the conventional model glutamine substrate, Z-QG, has been synthesized. Fluorescein-4-isothiocyanate-β-Ala-QG was shown to be an effective glutamine substrate for MTG for reaction with a lysine-containing peptide tag (dubbed as a “K-tag”), genetically encoded at the *N*-terminus of the peptide or protein of interest [82][83]. This K-tag was six amino acids in length, and both the second and fourth residues were lysines (MKHKGS). Mass spectrometry revealed that MTG displayed a high preference for the second lysine. The same group later developed two 13-mer peptidyl loop K-tags, each containing a single lysine, specifically recognized by MTG [84]; no direct comparison of the reactivity of the 6-mer and 13-mer tags was conducted. The 13-mer tags were encoded into bacterial alkaline phosphatase (BAP), which had been selected because MTG does not recognize any of its native glutamine or lysine residues as substrates. High labeling yields (>94%) were obtained when the 13-mer tags were inserted in vicinity of the active site, or at a location distal from the active site (Figure 6A). However, insertion distal from the active site provided higher reactivity. The reactivity of the two 13-mer tags was comparable. Using a different approach, incorporation of a fluorescent substrate was observed by an intramolecular FRET between two fluorescent substrate proteins, allowing an evaluation of transamidation activity of TG2 [85]. With this assay, propargylamine was found to be an excellent substrate for TG2. Following propargylation of a glutamine residue in casein, the resulting alkyne-modified residue was fluorescently labeled through a copper-catalyzed Huigsen cycloaddition with an azido-fluorescein conjugate (click chemistry) [86], thus providing a general route for labeling with a variety of azido-containing compounds. MTG was also found to be capable of using propargylamine as a substrate; additionally, it can use amino azides as substrates, to allow ulterior click chemistry with a variety of alkyne-containing compounds [63]. The techniques above offer high reactivity *in vitro*; however, they have not yet been tested in the context of cellular visualization.

TG2 is associated with tumor growth and drug resistance, but attempts to detect TG2 in tissues can often be plagued by false positives. Magnetic resonance imaging is a powerful diagnostic tool, and TGase may in the future be detected in tumor cells by using a new contrast agent [87] containing a primary amine, designed so that it would serve as a substrate for MTG (Figure 6B). Upon cross-linking the agent onto a tumor, a MRI signal is created. Called chemical exchange saturation transfer (CEST), a particular proton signal associated with the CEST agent is selectively saturated, and the proton remains in exchange with surrounding water molecules. As a result, the MRI signal from the water surrounding the CEST agent is reduced, allowing for its location to be determined. The signal generated before and after cross-linking of the contrast agent differs, allowing for easy differentiation between the two species. Once again, this work remains at the level of *in vitro* experimentation in a model system and has yet to be tested *in vivo*. TGase-mediated labeling has also been further expanded to label biological macromolecules other than proteins, such as DNA and RNA [88,89] (Figure 6C). Nucleic acid hybridization techniques make it possible to detect the expression pattern of a particular gene, which may be indicative of a disease. *In situ* hybridization (ISH) requires binding of a target DNA sequence to a probe, followed by detection with radioisotopes, fluorophores, or antibodies. In a new hybridization procedure dubbed transglutaminase-mediated *in situ* hybridization (TransISH), a Z-QG-labeled DNA-peptide conjugate was synthesized using DNA primers containing Z-QG-dUTP. The labeled DNA can then be denatured and cross-linked to alkaline phosphatase (AP) containing a K-tag in a process mediated by MTG. The DNA-linked AP will then dephosphorylate 5-bromo-4-chloro-3-indolyl phosphate, leading to the development of a blue chromophore. The same concept was also applied to mRNA [90]. As additional detection is not required with TransISH, it simplifies common ISH protocols by bypassing these steps, allowing direct staining after washing the unhybridized probe.

Fluorescent tagging has also been performed using TG2 activity in order to monitor cellular processes as well as the implication of TGases themselves in disease. Click chemistry was employed in a clinical context to monitor native TG2-mediated protein serotonylation (TPS). With little discrimination with regard to its protein substrate, this process involves TG2 cross-linking of serotonin to glutamine residues, and is implicated in necessary biological processes as well as disease [91] [92]. A modified analog of serotonin, propargylserotonin, was synthesized so that it could react with azide-functionalized substrates and enhance the understanding of Ras and its role in previously unknown processes [93]. In addition, of clinical relevance, TG2 is known to play a role in fibrosis and vascular calcification. In order to probe this further, mechanism-based fluorescent inhibitors were designed to covalently label TG2, to investigate how its activity may relate to stiffening of arterial tissues [94]. 

## 7. Conclusions

Notable progress has been made in both fundamental and applied research of TGases, although many challenges remain. New efforts in engineering their production have been made, with recent biophysical studies supplementing the knowledge base on the enzymes. However, despite recent work with respect to engineering TGase towards new and different capacities, the goals and results remains largely conservative. Better understanding and characterizing the substrate specificity remains a prime interest so that TGase can be effectively applied in existing and for novel applications. The enzymes have also increasingly become a tool to accomplish new feats in biotechnology. New methods have been developed for detecting and quantifying TGase activity, allowing for increased sensitivity and even *in vivo* assessment. TGases’ natural ability to use protein and peptide substrates gives them potential to label target proteins or peptides, but is limited by its specificity. Some of the techniques discussed in this review have found ways to work around this limitation, however, many remain at the level of proof-of-concept, leaving room for further development and optimization. 
